# Review: Sugar beets as a substitute for grain for lactating dairy cattle

**DOI:** 10.1186/s40104-017-0154-8

**Published:** 2017-03-01

**Authors:** Essi Evans, Ulrike Messerschmidt

**Affiliations:** 1 Technical Advisory Services, Inc, 64 Scugog St, Bowmanville, ON L1C3J1 Canada; 2 KWS SAAT SE, Grimsehlstr. 31, P.O. Box 1463, D-37555 Einbeck, Germany

**Keywords:** Dairy cattle, Feed beets, Fodder beets, Methane, Sugar, Sugar beets

## Abstract

Dairy cows are customarily given grains and highly digestible byproduct ingredients as additions to forage to support milk production. In many parts of the world growing seasons are short, and the grain crops that can be grown may not provide adequate yields. Sugar beets, on the other hand are relatively hardy, and dry matter yields surpass the yields of most grain crops. There are however, perceptions that beets may not be suitable as a feed ingredient due to the fact that the storage form of carbohydrate is sugar rather than starch. With little analytical support, sugar has been rejected in many feeding programs with the view that sugar reduces rumen pH, fiber digestion and microbial yield. This review explores available facts revolving around these concerns. Information regarding the feeding of sugar beets is provided and the use of sugar beets as a partial replacement for grain is proposed.

## Background

Feeding sugar beets to dairy cattle to replace a portion of the grain in the ration is a concept that has not received sufficient attention. Sugar beets are noted for their storage carbohydrate being in the form of sucrose. Sugar beets can be grown in rotation with barley, wheat, beans, corn, rapeseed/canola, potatoes or pulses, and traditionally are processed for table sugar. This crop can grow in a wide variety of soil types, but grows best in sandy loamy soils with a pH of 6.0–8.0 [1].

Being roots, rather than seeds, sugar beets offer several advantages over the traditional high energy grains that are used in dairy cattle feeding programs. Katerji et al. [2] described the sugar beet as a deep root, able to tolerate both drought and high soil salinity through the ability of this plant to rapidly adjust to changes in osmotic pressure. The typical growing season is between 140 and 160 d, but can extend to up to 200 d [3]. Unlike grains, where seed yield is susceptible to environmental damage during different growth stages, with sugar beets, the storage root is harvested, and is much less prone to climatic anomalies (4). Furthermore, while the interruption of growth in grains from incidences such as early frost, drought or flooding, may result in a complete loss of the harvest, with sugar beets as a non-maturing crop, there is generally at least a portion of the crop remaining [4, 5].

Sugar beets are generally grown in the temperate zones, from latitudes ranging from 30 to 60° [5, 6]. Sugar beets are also grown in arid, semi-tropical locations due to their tolerance for high sodium and high alkaline soil, with commercial yields as high as 80 t/ha [3]. A number of studies have confirmed that sugar beets are relatively insensitive to changes in temperature. In a German report, while the optimum mean temperature for beet growth was found to be 18 °C from sowing to the end of June, by harvest in October, the differences had disappeared [7]. Yields of sugar beets and sugar content of the beets planted in five temperature-diverse regions of Greece did not differ by harvest [8]. However, Wahab and Salih [3] determined that yields are highly dependent on the availability of water. Over the two years of their study, yields averaged 66.9 t/ha with weekly watering, but declined to 35.0 and 24.0 t/ha when the crop was watered every two and three weeks, respectively.

Table 1 provides yield data for the crop years 2010 through 2015. When converted to a dry matter (DM) basis, the average yield/ha exceeds that of corn and other grains typically used in rations for cattle. In addition, according to the USDA-ERS for the years 2000 through 2007 [9–11] inclusive, the cost of production/ha averaged US$ 936.30 for corn, but only marginally higher (US$974.10) for sugar beets. With the higher DM yields, sugar beets would be expected to provide a considerable cost of production advantage ($ 74.25/t DM) over corn ($ 110.39/t DM). Indeed, Haankuku et al. [12] in a very thorough analysis demonstrated that sugar beets are a more economical source of fuel for ethanol production than corn bases on a study for the state of Oklahoma, United States.

**Table 1:** Average yield based on US Census reports for crop years 2010–2015 [9, 10]

	Yield, metric tonne/hectare	Dry matter, %	Dry matter yield, metric tonne/hectare
Corn^a^	9.64	88	8.48
Barley^b^	3.76	88	3.30
Sorghum^c^	3.97	88	3.49
Sugar beets^d^	62.50	21	13.12

^a^168.0 bushels/acre at 25.45 kg/bushel

^b^68.2 bushels/acre at 21.81 kg/bushel

^c^76.4 bushels/acre at 25.45 lb/bushel

^d^30.8 imperial tons/acre

Like corn, sugar beets can be fed in a variety of forms [13]. Sugar beets can be stored fresh for up to 180 d, with minimal loss in sugar content, depending on the climate conditions. Longer storage times for fresh beets can result in loss of sugar due to respiration. Sugar beets may also be ensiled, either alone or with other ingredients such as forage or grain. Gilbery et al. [14] successfully ensiled fresh sugar beets alone, as well as with alfalfa hay, dry rolled corn grain, wheat middlings, and wheat straw. Beauchemin [15] ensiled chopped beets with barley straw to achieve a silage product similar to whole-crop barley silage. Beet silage can also be prepared from whole crop beets, which consists of the beet root and the beet top [16].

### Nutrient composition and digestibility

Sugar beets used for feeding purposes are often called “feed beets” to differentiate them from fodder beets. Although using sugar beets to supply energy for dairy cattle is a somewhat new concept, using sugar beet residue and fodder beets in rations for ruminant animals is not new, and some perspective can be gained from research available for these two ingredients. Beet pulp is a common ingredient in cattle rations in many parts of the world, serving as an energy source, with no known or reported antinutritional factors. Fodder beets are a component of many ruminant diets in use in Europe and Australasia. The nutrient profiles of beet pulp, fodder beets and sugar beets are given in Table 2. As these data show, sugar beets are similar in nutrient composition/kg DM to fodder beets, but have a slightly greater DM and sugar content.

**Table 2:** Nutrient composition of beet pulp and beet roots (g/kg dry matter, unless otherwise stated)^a^

	Beet pulp		Beet root	
Nutrient	Dried	Fresh	Fodder	Sugar
Dry matter, g/kg	892	241	169^b^	236^b^
Crude protein	93	86	71^b^	61^b^
Neutral detergent fiber	481	493	124^b^	125^b^
Acid detergent fiber	241	248	59^b^	61^b^
Ether extract	9	5	4^b^	9^b^
Ash	77	68	53^b^	44^b^
Calcium	15.5	12.8	11.0	2.5
Phosphorus	1.0	1.1	3.7	2.3
Potassium	4.5	4.2	30.0^c^	15.2
Sodium	0.7	0.5	4.1^c^	2.5^d^
Magnesium	1.9	1.5	1.8^c^	2.0^d^
Starch	5	–	1	–
Sugar	76	51	725	760^e^
ME, ruminants, MJ/kg dry matter	11.2	11.2	11.6	11.9
N digestibility ruminants, g/100 g N	69.0	69.1	61.3^b^	56.7^b^

^a^All values from Feedipedia.org [17] Unless otherwise indicated

^b^Hartnell et al. [81]

^c^Bayerische Landesanstalt für Landwirtschaft [82]

^d^Feedbeets.com (13)

^e^Evans et al. [50]

On a DM basis, sugar beets contain approximately twice the calcium and less phosphorus when compared to grains such as barley or corn [17]. Normally, beets are cleaned of external soil, but if this step is omitted, ash levels will increase depending upon the amount of soil that may remain on the beets [18].

The importance of gaining a perspective regarding the feeding value of beet pulp for this discussion resides in the fact that beet pulp is the residue from the extraction of sugar from the sugar beet, and can be regarded as the sugar beet without the sugar component. As Table 2 shows, beet pulp is largely composed of neutral detergent fiber (NDF). This NDF is unique in that it has been shown to feature a very high cation exchange capacity [19], which tends to promote the maintenance of pH and a more stable rumen environment. Beet pulp NDF, is low in lignin [20] and several studies have reported that the NDF fraction is highly digestible. Getachew et al. [21] reported a 24 h NDF digestibility of beet pulp at 76 g/100 g NDF, and an overall DM digestibility of 90 g/100 g. Voelker and Allen [22] conducted a study to profile and evaluate pelleted beet pulp as an energy source. Beet pulp was substitutes for corn grain, and provided to lactating cows at 60, 120 and 240 g/kg DM of the dietary DM. No other changes were made to the diets. The NDF content of the diets rose from 243 g/kg DM for the control to 316 g/kg DM for the diet containing the greatest beet pulp proportion (240 g/kg DM). Likewise, starch declined from 346 g/kg DM for the control diet to 184 g/kg DM for the diet with 240 g beet pulp/kg DM. There were no differences in milk yield or fat corrected milk (FCM) yield that could be associated with levels of beet pulp tested. Feed efficiency increased marginally with the inclusion of beet pulp in the diet (Table 3). Thus, with sugar digestibility estimated to be close to totality [23], one would expect the digestibility of sugar beet DM to be very high, and again, supporting this ingredient as one that might be useful as an energy source for rations for dairy cattle.

**Table 3:** Cow performance with diets containing beet pulp substituted for high moisture corn^a^

	Beet pulp, g/kg of dry matter				
	0	60	120	240	*P* - value
Milk yield, kg/d	36.4	36.6	35.9	35.4	0.58
3.5% Fat corrected milk, kg/d	37.4	38.4	38.0	36.8	0.20
Dry matter intake, kg/d	24.8	25.0	25.1	22.9	0.11
Fat corrected milk/dry matter intake	1.51	1.54	1.52	1.62	0.05

^a^Voelker and Allen [22]

### Feeding sugar to dairy cattle

The carbohydrate component of the ruminant diet consists of a number of fractions with differing properties. Sugars are the least complex, followed by starches, pectins and then by the insoluble fibrous cell wall material. Likewise, there is considerable variability within each category with respect to rate and extent of degradation and fermentation end products. Lanzas et al. [23] as a component of the Cornell Net Carbohydrate and Energy System applied rates of 0.40/h for the degradation of sugars (including beet molasses) 0.10–0.35/h for starches, 0.08–0.40/h soluble fiber, and under 0.10/h for cell wall fiber. The differences in rates between sugar and starch were narrower than in older nutritional models. Furthermore, sugars captured within a cellular matrix may potentially be degraded more slowly than free sugar added to the diet per se.

Sugars can be available in the form of monosaccharides, such as glucose, galactose, and fructose. Sugars added to diets are often disaccharides with sucrose, lactose and maltose being the most common. These sugars are most often added to diets to improve ration palatability. Nombekela et al. [24] conducted an elaborate series of studies to assess the preference of cows for sweet, sour, bitter or salty. Of six cows, four preferred the sweet diet (15 g/kg added sucrose) as compared to the control diet. The control diet was preferred over the salty, sour or bitter flavored diets. When cows were allowed to choose between all diets, these researchers found a 59% probability that cows would choose the sweet flavored diet. This is in agreement with Forbes [25] and Provenza [26] as ruminants generally prefer feedstuffs with a sweet taste.

With the established relationship between sugar and palatability, many studies have been conducted to assess the optimum amounts of sugar needed to maximize dry matter intake (DMI) in dairy cattle feeding programs. Broderick and Radloff [27] conducted two such studies. In the first, past-peak lactation Holstein cows were given diets with dried molasses to increase the sugar content from 26 to 42, 56 and 72 g/kg DM. Molasses dried onto soybean mill feed served as the source of sugar, replacing high moisture corn in the experimental diets, so that the energy content of the diets changed only marginally. The researchers reported linear increases in DMI, acid detergent fiber (ADF) digestibility, and NDF digestibility, but no differences in 3.5% FCM or body weight (BW) gain. In the second feeding trial, liquid molasses replaced high moisture corn in diets for Holstein cows in peak lactation at the start of the trial, with the diets supplying 24 (control), 49, 74 and 100 g/kg DM of sugar. Dry matter intake increased with the diet containing 49 g/kg sugar, but DMI for the diets containing 74 and 100 g/kg of sugar did not differ from the control. There were no differences in 3.5% FCM or BW gain associated with the dietary treatments. The authors concluded that 50 g/kg DM of sugar was optimal when molasses was used as the supplemental sugar source.

In a follow up study, Broderick et al. [28] evaluated the addition of 25, 50 and 75 g/kg DM sucrose as a replacement for corn starch in diets that contained 600 g forage/kg DM for cows in early lactation when the trial was initiated. There were linear increases for DMI and milk fat yield as sucrose increasingly replaced corn starch in the diet. Ammonia nitrogen (N) in the rumen was reduced along with the efficiency of N use in the rumen with the additional sugar in the diet.

Several more experiments where sugar has been substituted for a grain source demonstrate that sugar can be used to partially replace grain. Sannes et al. [29] substituted 32.1 g/kg sucrose for ground corn in diets for dairy cows in mid lactation. There were no differences in milk production, milk composition or DMI that could be attributed to the inclusion of sucrose in the diets. Similarly, McCormack et al. [30] saw no differences in milk production or DMI when 50 g/kg DM sucrose was included in the diet. Penner and Oba [31] found that milk production was not reduced when they replaced corn grain with 47 g/kg DM sucrose in diets for cows in early lactation.

In the above studies, the concentrations of sugar added in diets have been modest, as a portion of the total non-fiber carbohydrate (NFC), with most of the NFC still derived from starch. The primary objection to feeding sugar in larger amounts is the perception that the sugar will ferment to acids quickly, lowering rumen pH and contribution to sub-acute rumen acidosis (SARA). There are in fact indications that such rapid fermentation of sugar can reduce rumen pH. For example, Golder et al. [32] dosed heifers that had been starved of feed for 14 h with a mixture of fructose (4 g/kg BW) and grain (8 g/kg BW) or grain alone at 12 g/kg BW. Rumen pH was lower with the fructose sugar in combination with grain than with the grain alone (6.5 vs. 6.7). Kim et al. [33] also saw modest reductions in rumen pH with added sugar, but again circumstances were quite extreme. The researchers infused 150, 300 or 450 g of sucrose in the rumen of sheep given 680 g silage DM/d. The silage was divided in 24 equal increments and offered hourly. Rumen pH declined from 6.90 on the all forage control diet to 6.67, 6.69 and 6.47 with the addition of 150, 300 and 450 g of sucrose, respectively, to the feeding program. In another study [34] cows receiving 5.3 kg of DM (consisting of 700, 240 and 60 g/kg of grass silage, barley grain and rapeseed meal, respectively) were supplemented with 1 kg of sucrose. The sucrose was supplied either in two increments, in two increments with sodium bicarbonate (0.25 kg/d) or infused throughout the day. Rumen pH declined the most with the two daily increments, falling from 6.28 to 6.03. The decline was lessened when the same amount of sugar was infused over 24 h (6.12) and did not change when bicarbonate was included along with the sugar in two daily allotments (6.24).

Such procedures may not, however, replicate normal feeding circumstances. Sugar would more likely replace a source of starch, rather than be added on top of the normal feeding plan, or serve as an energy source when an adequate supply of NDF was available. As well, animals would be eating throughout the day in most circumstances. De Vega and Poppi [35] provided sheep with diets that contained 0:100, 15:85, 30:70, 45:55 and 60:40 sucrose: low quality hay (790 g/kg NDF). Dry matter intake averaged 34% greater across all sucrose-supplemented diets, significantly increasing fermentation end products in the rumen. However, rumen pH was significantly lower than the control only with the highest sugar inclusion level (6.46 as compared to 7.21 for the control treatment). Huhtanen et al. [36] provided cattle with diets containing barley, sugar beet pulp with or without molasses (sucrose) substituted for a portion of the concentrate. All diets provided 470 g/kg of forage on DM basis. The molasses made up 170 g/kg of DM in the two molasses containing treatments. There were no differences in rumen pH that could be attributed to the diets. Chamberlain et al. [37] added 200 g of sucrose, lactose, xylose, wheat starch, or fructose to a basal diet consisting of 4 kg of grass silage to sheep (100 g twice daily). Relative to the forage control diet, xylose, starch and fructose reduced rumen pH. Sucrose and lactose did not reduce pH relative to the high forage diet.

In a more recent study, Penner and Oba [31] provided dairy cows with diets containing 47 g/kg added sucrose, replacing an equal amount of corn grain for the first 4 weeks of lactation. Rumen pH was measured every 30 s for a 48 h period at the end of each week of lactation. Rumen pH was significantly higher with the diet with added sugar than with the corn diet, even though a greater portion of the carbohydrate was fermented in the rumen. The researchers speculated that a greater portion of the carbon from the sugar may have been used for rumen microbial protein (MP) synthesis, resulting in a reduced acid load. Similarly, Martel et al. [38] replaced 0, 25 or 50 g/kg DM from corn grain with molasses. Rumen pH was higher with the higher sugar (molasses) diets, which contributed to higher milk fat yield. In a second trial comparing no added molasses to 50 g/kg DM molasses, total volatile fatty acid (VFA) concentrations were lower with the diet containing molasses than with the control, which again might indicate greater MP synthesis and therefore higher rumen pH with the more fermentable diet. In a review on feeding sugar, Oba [39] concluded that replacing starch in the diet with sugar does not alter rumen pH. Although there are extreme circumstances where pH may decline to a greater extent when sugar replaces starch in the diet, such as when feeding levels are restricted, it would seem sugar is less likely to contribute to lower rumen pH than may currently be believed.

Another major reason that reduced pH was cited as a concern is the relationship that has been established between lower rumen pH and depressed NDF digestion in the rumen. When the non-structural carbohydrate (NSC) content of the diet is increased, rumen pH is often depressed. This may occur with the addition of sugar, not unlike starch in such situations. For example, when Kahali and Huhtanen [40] added 1 kg of unbuffered sugar to the diet, rumen NDF digestibility fell from 748 g/kg to 684 g/kg in sympathy with the decline in pH reported in a companion paper [34]. Moreover, results for NDF digestion for diets containing sugar are mixed, even in trials reporting no change in rumen pH. Huhtanen [36] found that sugar from molasses as a partial replacement for either barley or sugar beet pulp did not alter rumen pH. However, rumen, but not total tract NDF digestibility declined with the diets containing molasses. There were no differences between diets with respect to rumen pH and rumen concentrations of VFA when sugar was elevated in diets ranging from 26 to 72 g/kg of diet DM [27], and there was actually a linear increase in NDF digestion in that trial. NDF digestion was unaltered in the trial of Penner and Oba [31] while rumen pH increased with substitution by sugar. In a continuous culture study [41] 75 g/kg NFC was added to diets in the form of 0, 25, 50 or 75 g/kg sucrose, with the remainder of the mix consisting of starch. NDF digestibility values were numerically, but not statistically lower for the cultures to containing 25 and 50 g/kg sucrose than for the sucrose-free control. However, NDF digestibility for the culture containing 75 g/kg of sugar was found to be statistically greater than for the control. These results show that sugar may not reduce NDF digestion when it is substituted for starch, but may reduce NDF digestion when added on top of an existing feeding regimen. Furthermore, changes in NDF digestion may occur with the substitution of sugar for starch or grain, and this may be unrelated to rumen pH.

Glucose supply can be critical for dairy cows in early lactation, and there has been some concern that replacing starch with sugar can lower the availability of glucose precursors [39]. Larsen and Kristensen [42] provided cows in early lactation diets in which 405 g/kg high rumen escape starch from sodium hydroxide treated wheat was replaced by fodder beets. The wheat-based diet provided 50 g/kg sugar, as compared to 284 g/kg sugar for the diet based on fodder beets. Plasma glucose concentrations were significantly lower when measured at 4, 15 and 29 d in milk (DIM) with the high sugar diet. Energy corrected milk yields were not statistically different, but were numerically lower at 15 and 29 DIM for the cows receiving the fodder beet diet. Plasma ketones, measured as beta hydroxybutyric acid (BHBA) concentrations were significantly higher with the diet containing fodder beets, but there were no cases of ketosis in any of the cows employed in the study. Penner and Oba [31] similarly witnessed higher plasma BHBA concentrations when cows received diets containing 87 g/kg sugar than when the diet provided 45 g/kg sugar.

The changes in plasma BHBA and blood glucose found in these two studies similar to findings obtained when butyrate is infused into the rumen. Huhtanen et al. [43] reported that plasma BHBA was elevated, and plasma glucose declined when butyrate was infused in the rumen of lactating cows. Infusion did not reduce milk volume, but did result in greater milk fat yield. Interestingly, Penner and Oba [31] reported no differences in rumen VFA production or VFA profile, and could provide no explanation for the greater concentrations of BHBA in blood when sugars are provided. The higher BHBA levels do not appear to reduce productivity when early lactation cows are provided with diets containing added sugar, but the underlying cause for the increase in blood ketones remains to be determined.

Another objection to providing sugar in diets is the perception that microbial growth and therefore MP yield will be reduced. The early work of Chamberlain et al. [37] using sheep showed that the supply of microbial N to the small intestine was 10.2, 14.8, 14.3, 13.1, 11.9 and 13.7 g/d for the all forage control diet, sucrose, lactose, xylose, starch and fructose treatments respectively. In that study, microbial N yield with sucrose was significantly greater than that with starch. Using an in vitro system, Hoover et al. [44] determined that the amount of microbial N produced depended on the amount of total NSC in the diet, rather than the amount of sugar per se. In another experiment, 75 g/kg DM of starch was replaced incrementally by 0, 25, 50 and 75 g/kg of sucrose [28]. There were no changes in MP yield, when calculated by abomasal purine appearance, or by the excretion of purine derivatives in urine. Khalili and Huhtanen [34] reported higher MP yields when sugar was added to the diet. However, bacterial N yield/kg organic matter digested in the rumen did not change with the diets examined.

Few studies have involved the feeding of high concentrations of sugar in replacement for starch in the diet. Ingredients such as molasses, whey, and citrus pulp contain sugar, but limiting factors for dietary inclusion may be nutrients other than sugar, such as minerals in molasses and in whey and soluble fiber in citrus [45]. Baurhoo and Mustafa [46] provided lactating dairy cows with diets containing either 30 or 60 g/kg DM as liquid molasses, in replacement for corn grain. There were no differences in energy corrected milk; however, milk protein yield declined with level of molasses, while milk urea nitrogen (MUN) increased.

Dried whey permeate was used to replace starch from corn or barley with lactose in a recent study conducted by Chibisa et al. [47]. The test diets had 47.5 g/kg more sugar than the controls, and reduced starch by the same increment. There were no differences in DMI, milk production or milk composition that could be associated with changes in the sugar content of the diet. In another study involving a product based on lactose, the product was exchanged for corn at the rate of 86 g/kg of the diet DM, with no decrease in milk production or DMI [48]. Milk fat was elevated by the sugar product. In an older study, Casper and Schingoethe [49] provided lactating cows with diets based on corn, barley or dried whey. The dried whey was included in the test diet at 15% of the total DM. Cows produced significant less milk (31.2 kg) with the whey diet than with the diet where corn was the primary carbohydrate (32.8 kg). In an experiment with mid-lactation dairy cattle, Evans et al. [50] fed cows diets that contained up to 191.2 g/kg DM as sugar from sugar beets, replacing starch from corn and barley. There were no differences in DMI or lactation performance (Table 4) in this study.

**Table 4:** Performances of cows in mid lactation given diets containing sugar beets substituted for corn and barley grain^a^

	Feed beets, g/kg of Ration dry matter				
	0	80	160	240	*P* - value
Sugar, g/kg dry matter	46	105	154	191	
Dry matter intake, kg/d	25.1	25.0	25.2	24.5	0.79
Milk yield, kg/d	26.6	26.0	26.4	26.4	0.83
Fat, %	4.64	4.66	4.72	4.72	0.95
Protein, %	3.54	3.44	3.47	3.44	0.59
Energy corrected milk, kg/d	30.1	30.4	31.1	31.2	0.73
Energy corrected milk/dry matter intake	1.25	1.23	1.25	1.28	0.69

^a^Evans et al. [50]

In summary, beets can be viewed as a mixture of beet pulp and sucrose. Inferences regarding response to beets in ration formulations can be obtained by reference to the research conducted on both accounts. Rates of degradation of sugars overlap with starch digestion rates for many commonly-fed grains, and hesitation to feeding beets on the basis of sugar content should not be any greater than the hesitation to feeding starch from rapidly fermenting grains such as barley or wheat.

### Syncronization of nutrients in the rumen

The rumen is a significant source of nutrients to the animal. Much of the protein and amino acid requirements are met through the synthesis of the microbial biomass. Additionally, microbial production is responsible for the degradation of cell wall fiber, and the production of VFA for utilization by the animal. Depending on the level of production of the animal, the rumen may provide a substantial portion of the animal’s nutrient supply.

Obviously, optimization of the performance of the rumen would be of great benefit in improving efficiency and animal productivity. Numerous studies have been conducted in attempts to synchronize the rumen: provide carbohydrate fractions and nitrogen fractions with similar rates of degradation in relationship to each other. The theory is that by supplying energy and nitrogen sources in the rumen concurrently an increase or optimization of microbial efficiency (g MP/g substrate) should occur.

Attempts to synchronize rates of nutrient digestion have been mixed. Chanjula et al. [51] saw no differences in microbe numbers, DMI or performance when cows were given two starch sources at two levels of inclusion, with and without added urea. Chumpawadee [52] used a synchrony index based on digestion rates of protein and NSC, and saw a tendency towards higher DM digestibility. Biricik et al. [53] found no difference in digestion or performance. Yang et al. [54] reviewed results from 18 studies. Microbial protein synthesis was numerically greater in 10 of the studies.

Illius and Jessop [55] pointed out that rumen metabolism cannot be considered in isolation. The influx of nutrients into the rumen is not derived strictly from the breakdown of ingested feed. Microbial recycling, hindgut fermentation, and blood ammonia all contribute to steady state conditions. Cole and Todd [56] considered both rumen and hindgut fermentation and suggested that oscillating dietary N levels could contribute to greater recycling from the blood and hindgut into the rumen, and would improve efficiency. According to Aschenbach et al. [57] short chain fatty acid absorption across the rumen wall increases the influx of urea N into the rumen, providing a supply of N to support microbial growth.

Hall and Huntington [58] delineated the numerous reasons why synchrony would be an illusion. These include, describing rumen metabolism, predicting post-ruminal supply of nutrients and their absorption, how the animal uses the absorbed nutrients as well as changes in patterns with altered states and changes in the environment. They note that a whole animal approach must be taken. Ruminants may also alter feeding patterns when diets are modified to contain high concentrations of sugar. In a study conducted in sheep [35], sheep consumed feed at a slower rate for the first 5 h after feeding as sugar content increased from 150 to 600 g/kg of DM.

As a final note, most models fail to account for the fact that cows eat more than one meal/d. As pointed out by Firkins [59] there is a large variation in meal patterns among cows and even by the same cow over multiple days. The more frequently the meals are consumed, the more this daily composite of dietary carbohydrate sources is divided into smaller increments, reducing any potential burst of fermentation from any given source. As a case in point, even under extreme conditions, constant intraruminal infusion of 450 g of sugar in sheep receiving only 680 g of DM/d failed to elicit an acidotic condition [33].

In conclusion, the need for nutrient synchrony for diets containing high amounts of sugar as a replacement for starch should not be of concern under most normal feeding situations. Data are available to support the fact that synchrony of carbohydrates and N is not necessary when feed is continuously available.

### Feeding of beets

Sugar and fodder beets are not as common energy sources as grains for dairy cattle, in spite of their apparent agronomic and economic virtues. The reason for the low acceptance may partially be due to a lack of familiarity with these two ingredients, as well as some of the special handling requirements. Matthew et al. [60] even went as far as to suggest that fodder beets are a problematic feed, and due to many similar features, sugar beets might therefore pose many of the same issues.

One concern with feeding beets is the low DM content. Eriksson et al. [61] provided cows with three diets where the concentrate portion of the diet consisted of (DM basis) an 80:20 mix of barley and raw potatoes, and 80:20 mix of beets and raw potatoes and lastly all barley. Milk yields with the three diets were 24.7, 23.0 and 25.3 kg, respectively. The corresponding DM contents of the diets consumed were 390, 280, and 420 g/kg. The researchers noted that less silage was consumed with the beet diet. These levels are considerably below the recommended DM range of diets for dairy cows. The NRC [20] recommendation for DM content of rations for dairy cows is above 500 g/kg to prevent reduced DMI. Current British recommendations indicate that the optimum DM content of rations should be in the range of 450–550 g/kg [62].

Methods to overcome the high moisture level of beets have been devised. Mixing with forage at ensiling is one technique that has been used to advantage. Bell et al. [63] added 100 g/kg beets on fresh weight basis to wet corn forage (180 g/kg DM). Milk yield, fat yield and protein yield were higher with the mixed silage than the corn silage alone. Another approach would be to use sugar beets to partially replace grain, while maintaining ration moisture content within the prescribed range. Both methods, however, limit the inclusion of fodder beets or sugar beets in the diet.

Another reason performance with beets may be lower than expected might be incorrect formulation. Mogensen and Kristensen [19] replaced barley with beets in a total-mixed ration. Energy corrected milk was 1.4 kg lower with the fodder beet diet. The beets used in the study contained 210 g/kg ash on a DM basis, and this was not taken into account when the diets were formulated. Normally the ash concentration of sugar and fodder beets is low, as indicated in Table 2 on DM basis. However, if the beets are not cleaned after harvesting, soil adhering to them will dilute the feeding value. Schwarz et al. [64] supplemented wet forage with beets, corn grain or a supplement containing protein. Milk production was higher with the beets (17.5 kg) than with forage alone (15.8 kg), but less than with corn (19.1 kg) or concentrate (19.6 kg). In this experiment, the beet diet only provided 130 g/kg total crude protein (CP) and it is likely that the low CP may have limited production. This is in agreement with a study by Fisher et al. [65] where supplementation with fodder beets (4 kg of DM/d) only improved milk production when higher levels of CP were supplemented. In similar fashion, Ferris et al. [66] calculated that energy intakes were higher with diets containing fodder beets mixed with silage than silage alone, but did not see any gains in milk. This suggests that the energy content of the beets was overestimated.

The availability of energy for microbial production is imperative to insure MP availability and to maximize rumen fiber digestion. Eriksson et al. [67] demonstrated that fresh fodder beets supported microbial growth to the same extent as fresh potatoes. Relative to silage, both energy sources reduced MUN, and roughly doubled the calculated amount of rumen MP produced.

Ferris et al. [66] compared grass silage or grass silage with 300 g/kg fodder beets on DM basis at five levels of concentrate allocation. The calculated energy value of the diets was not different at each level of concentrate. Dry matter intake was higher with diets containing beets than their counterparts without beets. However, there was no change in milk yield in this study. Total purine derivatives increased with the fodder beets, indicating enhanced microbial output with the diets that included beets.

There are several references that suggest that beets and/or sugar fermentation in the rumen increases the amount of methane produced therein, and could result in lower than expected available energy. On average, 8–12% of the dietary energy is lost in the rumen due to the production of methane by rumen microbes. In an older study [68] sheep were used to estimate the ME value of beets, and determined that the value obtained in their studies was about 10% less than would be calculated from chemical analyses, and implicated formation gases. Buddle et al. [69] advocated shifts in forages high in sugar from forages high in soluble fiber to reduce methane production, noting a relationship between sugar fermentation and gas production. Mills et al. [70] developed a model, where based on older data, sugars are presumed to ferment to acetate and butyrate, with methane the result, while starches result in a propionate fermentation and lower the production of methane.

Results from some studies, however, indicate that methane production may be more variable, depending upon rumen dynamics. Hindrichsen et al. [71] found that sugar increased the release of methane in fermenter studies. There were however, no differences in the production of VFA between diets containing sucrose added from sugar beet molasses (188 g/kg) and starch added from wheat (228 g/kg). Likewise, Erikkson and Murphy [72] showed in their fermenter studies that substrates, including beets, may be capable of supporting different populations of rumen microbes with different requirements and output potential. VFA production rates also depended on the diet and level of feeding of the rumen fluid donor animal. Thus, the proportion of energy given up as methane depends on an understanding of the diet and rumen conditions. Less methane is product when sinks are available to capture excess hydrogen. Unsaturated fat is an example [73]. Also the pH of the rumen has a great influence on the VFA and single carbon molecules that are generated. McAllister and Newbold [74] pointed out that all complex carbohydrate is converted to sugar prior to fermentation in the rumen, indicating that temporal differences may be important when measuring methane. In the conceptual model of Janssen [75] rate of fermentation and rate of passage interact in the growth of methanogenic organisms, and this model provides the best explanation of the differences that were found in previous studies and how they relate to study design as much as to methane generation. More research is needed to determine if methane production is greater with sugar beets than with grain in diets for dairy cows.

Oddly enough, there is evidence that the fiber in sugar beets may mitigate the rate of digestion of starch in the diet. Proving beet pulp rather than corn reduced the amount of starch, and increased the amount of fiber in the diet [76]. Interestingly, the study revealed that starch digestion shifted from the rumen to the intestine as beet pulp was added to the diet. Hatew et al. [77] confirmed a reduced rate of starch digestion with beet pulp in the diet. As beet pulp contains essentially no starch, this would strongly suggest that digestion of starch from other ingredients is changed with beet pulp added to the diet. Guo et al. [78] did not measure rate of starch digestion, but found that finely ground wheat produced less SARA when beet pulp was included in the diet.

A newer study [79] provided sheep with high forage diets (700 g/kg DM), where the test components included fructosan, inulin or sucrose (89 g/kg DM). Sucrose increased rumen propionate concentrations, while butyrate became elevated with fructosan and inulin, when compared to the control diet. Acetate concentrations were lower than the control with all the test articles.

Generally, the time spent chewing during eating and ruminating increased with fiber and as particle sizes increase. A study conducted by researchers in Belgium [80] indicated that the physical structure of beets resulted in high chewing times. Cows given fodder beets as compared to ensiled beet pulp spent more time chewing during eating (16.4 min/kg DM as compared to 10.5 min/kg DM). The total time spend during eating and ruminating were similar (32.3 min/kg for beet pulp and 34.3 min/kg for fodder beets), even though the beets contained much less fiber (116 g/kg NDF for beets and 448 g/kg NDF for beet pulp). This would be expected to increase the energy expenditure of cows to some extent, and could result in some loss in performance relative to values calculated from chemical analyses alone.

Evans et al. [50] provided dairy cows with diets that contained 0, 80, 160 or 240 g/kg of the total ration DM as fresh, chopped sugar beets. There were no losses in milk production, milk composition or DMI in this study when compared to the control ration in which the concentrate was based on corn and barley (Table 4). In this Latin Square study, diets were changed abruptly, and there were no times when cows were off feed.

These results appear to suggest that fresh beets might not pose a greater risk of digestive or metabolic upset than grains when presented as a portion of a total mixed ration. However, more studies are required to assess the effects of providing beets in early lactation, as well as feeding for greater lengths of time.

## Conclusions

To summarize, the sugar beet crop yields greater amounts of DM and energy than many common grain crops, and from that perspective appears to provide an advantage as a source of energy for dairy cows. There have been few feeding trials to adequately support the feeding of fodder or sugar beets as a partial replacement for grain in rations for high producing dairy cows. Part of the problem is a lack of up to date nutrient values in feed ingredient tables. The analytical results for the composition of beets supplied on the Feedbeets.com [13] website should provide great support for formulations, but needs to be verified by additional sources. Data regarding digestibility and availability of nutrients for current varieties are limited [81]. However, there are trusted values for digestibility of individual nutrients in beet pulp and can be used to extrapolate values to beets.

To date there have been no reported anti-nutritional aspects of feeding beets. Data regarding fermentation of sugar in the rumen seem to suggest that changes in rumen pH are not a major concern in cows fed multiple times/d; however, consideration should be given to results from studies that show that plasma BHBA can be elevated when sugar replaces starch in early lactation. The greatest obstacle to feeding fresh sugar beets to high producing dairy cows is the low DM (200–230 g/kg) content. For a typical diet containing 500 g/kg silage at 350 g/kg DM, sugar beet inclusion would need to be limited to 200 g/kg DM or less in order to insure that intakes are not compromised by too much moisture. Further research is needed to fully explore the potential cost savings of including sugar beets in dairy feeding programs.

## Abbreviations

ADF: Acid detergent fiber
BHBA: Beta hydroxybutryric acid
BW: Body weight
CP: Crude protein
DIM: Days in milk
DM: Dry matter
DMI: Dry matter intake
FCM: Fat corrected milk
MP: Microbial protein
MUN: Milk urea nitrogen
N: Nitrogen
NDF: Neutral detergent fiber
NSC: Non- structural carbohydrate
SARA: Sub-acute rumen acidosis
VFA: Volatile fatty acid
